# Analyzing the Characteristics of Policies and Political Institutions for the Prevention and Control Governance of the COVID-19 Pandemic: Evidence from China

**DOI:** 10.3390/ijerph191710980

**Published:** 2022-09-02

**Authors:** Mingniu Dong, Cheng Zhou, Zhenhua Zhang

**Affiliations:** 1 School of Public Administration, Nanjing Normal University, Nanjing 210023, China; 2 School of Marxism, Tongling University, Tongling 244061, China; 3 Institute of Green Finance, Lanzhou University, Lanzhou 730000, China

**Keywords:** COVID-19, public policy, political institution, effective governance, China

## Abstract

This study explores the institutional reasons for and logical mechanism of the Chinese government’s rapid positive results and major strategic achievements in coronavirus disease 2019 (COVID-19) pandemic prevention and control. Based on the ROST Content Mining System version 6.0 (ROST) and VOSviewer V1.6.1 (VOSviewer), we conduct an econometric visualization analysis of COVID-19 pandemic prevention and control policies to explore which strengths of Chinese political institutions have been brought into play by the Chinese government and how to systematically analyze the approaches by which these strengths support effective public governance. The findings show that: (1) “institutional strength”, “medical terminology”, “policy content”, “policy implementation object”, “policy implementation requirement”, and “policy-making and implementation actor” are the six groups of high-frequency keywords in prevention and control policies. (2) The occurrences, links, and total link strength of the seven Chinese institutional strength keywords are very high. These results mean that the Chinese government has made full use of its institutional strengths to prevent and control COVID-19. These findings indicate that institutional strengths are critical to public health crisis prevention and control. They also illustrate that institutional strength is the prerequisite and key factor for achieving effective governance in the policy process. Scientific policymaking, efficient policy implementation, and strict oversight are undeniably necessary for effective governance during public health crises.

## 1. Introduction

In the 21st century, public crises have frequently occurred. Public security crises (e.g., the 9/11 terrorist attacks, the Bali bombings, and hostage-taking situations in Moscow and Beslan), natural disaster crises (e.g., the Indian Ocean tsunami, Hurricane Katrina, and the Wenchuan earthquake), and production accident crises (e.g., the Fukushima nuclear leak and the explosions in the Beirut and the Tianjin ports) are typical public crises [1], and they affect social conditions, the economy, and people’s lives [2]. Likewise, public health crises should not be underestimated; SARS, Ebola, and the coronavirus disease 2019 (COVID-19) have affected the entire world and pose a great threat to the life, health, and safety of human beings [3,4,5]. The current crisis is transforming human society into a “risk society” with unprecedented breadth and depth [6]. The increase in public crises seriously threatens the safety of human life and health and undermines global economic and social development [7,8]. It has also repeatedly raised the need for public governance, poses serious challenges, and tests the effectiveness of governance in countries worldwide [9]. Public crises pose a serious threat to national governance goals and structures [10] and are characterized by tremendous time pressures and a high degree of uncertainty. Therefore, how to respond to and manage public crises has become a frequent and urgent issue for governments and a leading research subject among public governance scholars.

Governance in public crises varies by country depending on national conditions, geography, and ideology. However, the limitations of the “technical means of governance” are gradually emerging [11]. The modernization of the public crisis governance system and capacity needs to “find another way” by starting from the underlying factors that affect effective governance, that is, to find other effective institutional designs [12]. In terms of global public governance theory and practice, political institutions are important prerequisites for public crisis management and even for the whole field of public governance. Effective governance is a reflection of the effectiveness of the entire political process of “state institutions–policymaking–policy implementation” [13]. Political institutions do not work automatically, but they can translate into effective governance through a series of policy processes [14]. Therefore, efficient governance during public crises requires the clarification of policy approaches and logical frameworks based on political institutions [15], that is, how political institutions are embodied and applied in the policy process and how they are translated into effective governance [16].

Undoubtedly, the COVID-19 pandemic is a typical public health crisis, and its governance effectiveness also definitely depends on the political system and prevention and control policies. For China, scholars commonly agree that the fundamental reasons for the Chinese government’s rapid positive results and major strategic achievements [17] in COVID-19 pandemic prevention and control [18] and its system of policies established and implemented under existing institutions [19] are China’s institutional strengths. A few studies have considered institutions by often focusing on the governance institutions developed to resolve public crises rather than on the underlying factors that influence the development of governance institutions and institutional (State or political) choices [20]. However, few scholars have attempted to systematically and empirically study the characteristics of policies and political institutions for the prevention and control of the COVID-19 pandemic. Policy studies can help provide insight into governance practices in COVID-19 pandemic prevention and control [21,22,23]. Therefore, this paper takes the COVID-19 pandemic prevention and control policies in China as an example to study the approaches to achieving effective governance through political institutions.

Five sections follow this introduction. Section 2 reviews the literature on political institutions and effective governance. Section 3 describes the data source and the methodology. Section 4 presents the results of the econometric visualization of the COVID-19 pandemic prevention and control policies. Section 5 provides a discussion and implications for approaching effective governance through policy processes. Section 6 concludes the paper.

## 2. Literature Review

### 2.1. Political Institutions

The research on political systems is divided into two main streams. The first stream concerns the concept and connotation of political institutions, with paths of interpretation that tend to vary across different perspectives. The second stream concerns political institutions; these scholars explore whether and how political institutions affect the development of politics, the State, and society.

It is clear from the long evolution of political science that institutions have often been seen as central to the advancement of political science and national governance and are identified as the pivot and keystone of political science research and national governance studies. However, studies devoted to clearly explaining the concept and content of political institutions are relatively rare among the works of March and Olsen, Lane and Erskine, and other scholars, such as in the *American Political Science Review*, *Rediscovering Institutions: The Organizational Basis of Politics*, *Western European Politics and Society*, and *New Institutional Politics*. Although some scholars touch on [24] or outline [25] the concept of the political system, these discussions seem to be too brief and cause confusion [26], or the concept of the institution and the political science context do not sufficiently “resonate” [27] to present the real semantics and a profound connotation of the political institution [28].

Political scholars consider the political institution to be an inherently ambiguous concept or believe that structure and institution as concepts overlap and substitute for one another, as demonstrated by grand models such as the structural functionalism commonly referred to in the 1960s and 1970s. In his in-depth study of political institutionalization, Samuel Huntington, an American political scientist in the 1970s, proposed that “institutions are stable, valuable, and recurring patterns of behavior” [29]. Political institutions are political organizations and procedural arrangements that arise to resolve disputes over the different interests of a political community and to maintain a certain order [30]. Huntington’s definition appears in line with the classic definition of institutions proposed by the American sociologist Talcott Parsons in the 1950s: “the deep patterns of reasonably expected behavior of people when social structures require them to play their roles according to a script” [31]. It also coincides with French political scientist Duverger Maurice’s idea that “a political institution is the form of a particular social group distinguished by the ruler and the ruled” [32]. Huntington’s account of institutions and political institutions is also accepted by scholars such as Robert Goodin, who suggested that academic traditions show different tendencies in conceptual definition, which are somehow “internalized” in their respective processes of practice [33].

For classical institutionalists, who are represented by Montesquieu and de Tocqueville, an institution is closely related to the social group that drives political and economic development. In his book, *Considerations on the Causes of the Greatness of the Romans and their Decline*, Montesquieu wrote that two factors, namely, the political institution of the State and the customs of the inhabitants, were decisive in the rise and fall of the ancient Roman Empire [34]. Many long-established political theories have posited that “political science itself is a holistic discipline of knowledge that takes institutions as its research path” [35]. The analysis of complex political phenomena and effective governance through the origin, evolution, and mechanisms of political institutions has long been a hot research topic for scholars in the fields of political science and public administration [36]. There is a strong tradition of institutional analysis in political science, public administration, and sociology, as scholars have long recognized that the pursuit of national interests and civic values loses meaning once the institutional context is removed [37], and the merits of political institutions directly affect economic and social development [38] and the effectiveness of public governance [39]. Since 1990, the empirical studies of three new institutionalisms in political science—historical, rational choice, and sociological—have grown significantly in scope and explanatory power [40]. Bo Rothstein argued that “whatever story a political scientist tells, it is necessarily related to the political institution” [41]. These studies make it abundantly clear that political institutions influence public policy choices [42] and effective governance [43] and determine the extent of political, economic, and social development [44]. In addition, in fields including the choice of political institutions, scholars such as Peter Hall [45] and Desmond King [46] have argued that “perception” is an important factor in institutional choice and that different political and economic perceptions influence the choice of different political institutions. They have posited that institutions are vehicles for perceptions and that perceptions guide the development of states and societies by shaping organizations and individuals to perceive and choose in ways that are consistent with their preferences by providing a link between institutional structures and cognitive factors. Among other scholars, Vincent A. Ostrom believes that the formulation and choice of political institutions need to change from “monocentric” to “polycentric”, and he pioneers the concept of a “polycentric political institution” that advocates the collaboration of multiple actors, such as governments, social organizations, businesses, and individual citizens.

### 2.2. Effective Governance

Governance differs from traditional rule or public administration in that it provides the organizational framework for public administration [47], which implies a new way of ruling [48] and a new approach to public administration [49]. Governance achieves effectiveness by creating conditions to ensure social order and collective action [50]. Many scholars have devoted themselves to analyzing which factors affect effective governance. Commonly accepted factors are a plurality of governance actors: their efficient synergy and organic interaction, behaviors, institutions, and democratic governance.

For Kooiman and Van Vliet, governance is essentially a process of interactions among actors, in which multiple processes of domination influence one another [51], and its effectiveness is because of the interaction of various institutions involved in collective activities in a context of the interdependence of power. The interaction of various actors through interdependent, multi-structured political and social networks constitutes the empirical basis of modern governance, i.e., such interactions provide the logical preconditions for achieving effective governance [52].

Some scholars believe that, in addition to the interaction of various institutions or actor-effective governance, institutional interaction can achieve effective governance. Olav Schram Stokke—who proposed the idea of interactive management based on utilitarian, normative, and conceptual interactions—notes that interactive management is political governance in response to the effects of institutional interaction [53]. Thomas Gehring and Sebastian Oberthür believe that the multiple interactions of institutional obligations, behaviors, governance goals and public governance share a causal mechanism [54]. From the perspective of interaction, effective governance is the result of the interaction of governance subjects, behaviors, and policy institutions.

Interactive governance is also embedded in many theoretical governance paradigms, such as collaborative, democratic, and participatory. Collaborative governance focuses on the identification, recognition, and role determination of collaborative governance actors [55] and argues that the motivation for participation, rights and resource allocation, leadership, and institutional design of multiple collaborative actors are the key variables that affect the realization of effective governance [56]. Democratic and participatory governance intersect with one another. Democratic governance emphasizes the participation of stakeholders in the governance process to promote the realization of effective governance. The paradigm is gradually becoming one of the important political attributes of many political parties in power, which coincides with the connotation of participatory governance. Both aspects further promote democratic governance represented by citizens’ participation, which enhances effective governance and citizens’ value [57].

Other scholars have studied the interrelationship between policy science and effective governance directly. Harold D. Laswell, an American scholar, believes that the basic research paradigm of policy science is the pursuit of policy “rationality” and that once a policy has “rationality”, it can be translated into effective governance. He also emphasizes the empirical paradigm of policy research [58]. Lasswell’s logical positivism about policy science has become a dominant paradigm in the field of policy science and public governance [59]. An increasing number of scholars are exploring the deep relationship between policy science and effective governance through the physical carrier of policy. Thus, the econometric analysis of the policy literature that follows the “positivism” research paradigm of policy science has gradually become common in policy science and policy analysis in the field of public governance in recent years [60]. In this new paradigm, content, econometric, social network, and visualization analyses are used to quantify the content of public policy and reveal the characteristics of the proliferation and evolution of public policy themes, the combination and effects of policy tools, and the actions and relationships of policy actors to ultimately analyze the principles and factors that influence effective governance.

## 3. Data and Methods

**Figure 1:**
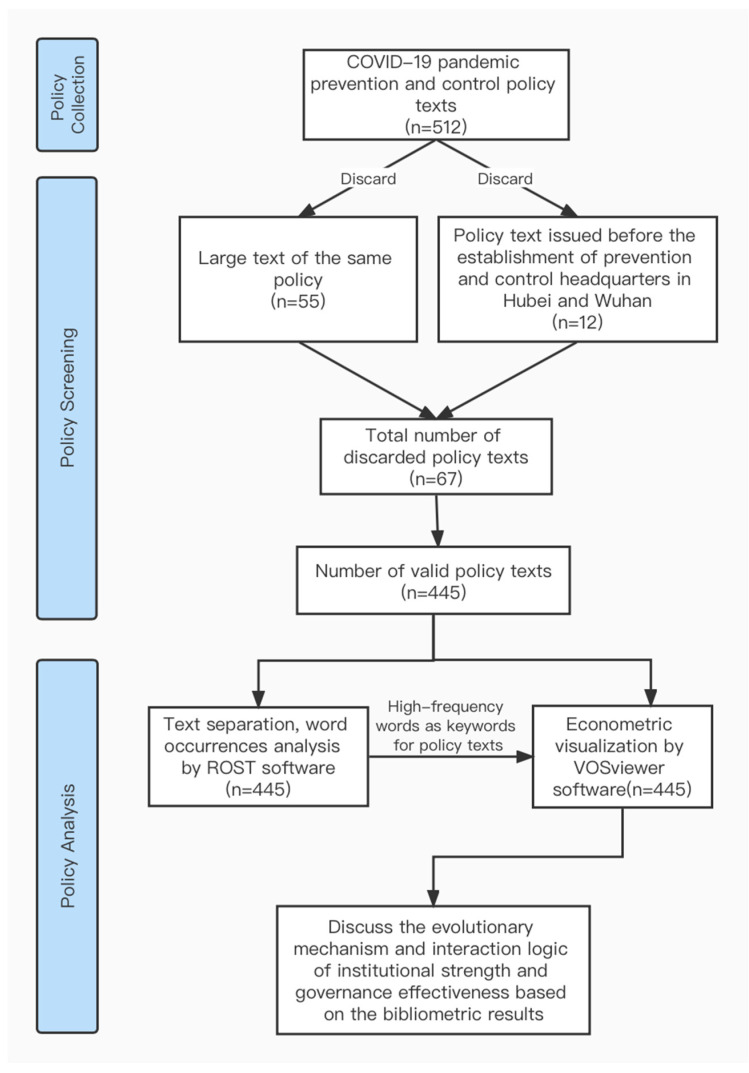
Data processing and research methodology. Source: Authors’ compilation.

By selecting a series of COVID-19 pandemic prevention and control policies in China, a policy bibliometric analysis is used to explore the institutional strengths that the Chinese government has capitalized on and how it has capitalized on them in the COVID-19 pandemic prevention and control process. Then, based on these institutional strengths and governance practices for COVID-19 pandemic prevention and control, we systematically analyze the approaches by which China’s institutional strengths are translated into effective governance in the context of public governance (Figure 1).

### 3.1. Data Sources

For this paper, we downloaded 512 COVID-19 pandemic prevention and control policies from the official websites of the central government, the Hubei and Beijing provincial governments, and the Wuhan municipal governments. We ultimately excluded 67 policies and retained 445 policies for a textual and econometric visualization analysis to outline the characteristics of prevention and control policies for the COVID-19 pandemic in China. Specifically, the data collection and processing were completed by following the two steps outlined below.

#### 3.1.1. Policy Scope Determination

To ensure the representativeness of the cases, we selected the regions in China with the most severe COVID-19 pandemic as of June 2020, which have the most released policies for pandemic prevention and control.

The first region is Hubei Province and its main city, Wuhan. Wuhan was the first city to find COVID-19 cases and was strongly affected by COVID-19 in China during the first half of 2020. The number of confirmed COVID-19 cases was 68,135, the rate of severe illness was close to 40%, and the mortality rate approached 6.62% in Hubei Province as of 27 May 2020, which was the peak of COVID-19 infection in China; these three figures are 3.15, 20.16, and 7.17 times higher, respectively, than those outside Hubei Province. The Chinese central government also sent a central steering group to Hubei and frequently mentioned “winning the battle of Hubei and Wuhan” in the prevention and control policies. Therefore, the series of COVID-19 pandemic prevention and control policies issued by the Hubei and Wuhan governments are the most representative and typical and have greater consistency, coverage, and inclusion than the policies of other provinces and cities.

The second region is Beijing. In June 2020, a new COVID-19 pandemic cluster outbreak occurred in Beijing, and 335 new cases were confirmed. As the capital of China, Beijing has significant importance and special characteristics in the prevention and control of the COVID-19 pandemic, and the ministries and commissions of Beijing and nationwide recognized this importance and formulated resolute, decisive, and strict prevention and control policies to contain the spread of the pandemic.

Finally, the Chinese central government also plays an important policy role in the prevention and control of the COVID-19 pandemic. Hubei and Wuhan had poor formulation and implementation of prevention and control policies, which showed problems such as low political standing, insufficient attention, and lack of awareness of responsibility in the early stage of the COVID-19 pandemic. Therefore, the Chinese central government intervened strongly and rapidly. At various important junctures in the prevention and control of the pandemic, the Chinese central government gave full play to its leading role and formulated a series of critical prevention and control policies in a scientific, timely, and targeted manner, which demonstrated the significant institutional strength of the centralized and unified leadership of the Communist Party of China (CPC) (Centralized and unified leadership refers to the Chinese central government of the CPC, which leads all the work of the country. Mao Zedong once said, “Concentrate all possible and necessary powers in the central government and central representative organs” to strengthen the authority and centralized and unified leadership of the CPC Central Committee).

**Figure 2:**
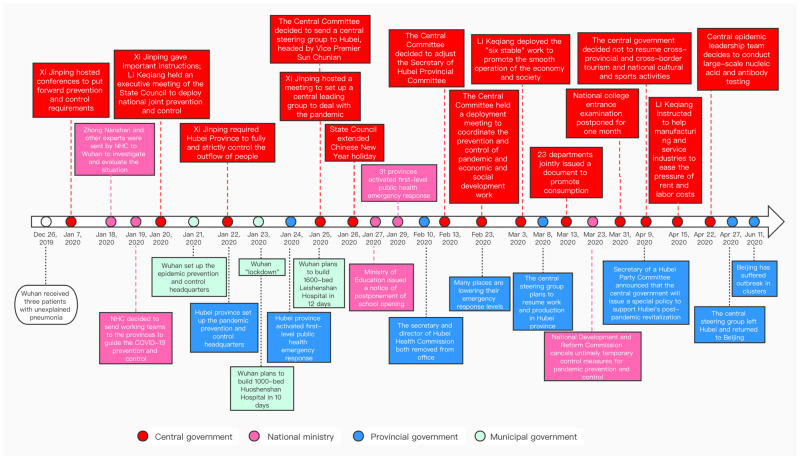
Timeline of China’s policy response to the COVID-19 pandemic. Note: Red, pink, blue, and green refer to the policies issued by the central government, national ministries, provincial government, and municipal government, respectively. Source: Authors’ compilation.

Consequently, this study selected the policies issued by Hubei, Wuhan, Beijing and the Chinese central government before 1 July 2020. The policy timeline of China’s response to COVID-19 (Figure 2) indicates that when the central, provincial, and municipal governments introduced prevention and control policies applicable to specific contexts, all played pivotal roles in the prevention and control of the COVID-19 pandemic.

#### 3.1.2. Policy Screening

After determining the policy scope, we downloaded all the publicly available policies until 1 July 2020 from the official government website, which totaled 512 policies. In terms of policy selection, we chose only policy documents that have clear prevention and control provisions, i.e., policies that clearly identify which measures should be used by different responsible actors to prevent and control the pandemic. For example, the *Notice on Strengthening Party Leadership and Providing Strong Political Assurance for Winning the Pandemic Prevention and Control*, issued by the Chinese central government, clearly stipulates that Party organizations and their members at all levels of the Chinese government are the most important actors in the prevention and control of the COVID-19 pandemic and that they should work together with different governmental departments to play their roles in medical rescue, scientific research, and elementary precaution. At the same time, in terms of the policy level, this article selects only the central government, provincial governments, and municipal governments because most of the important policies for pandemic prevention and control in China are made by municipal governments and above, which often have the most obvious policy influence and effectiveness.

First, through a comparative analysis of the policies formulated before and after the establishment of the headquarters for the prevention and control of the COVID-19 pandemic, we found that the most important and influential policies and key prevention and control measures of the Wuhan and Hubei governments were issued after the establishment of the corresponding headquarters. Therefore, we discarded 12 policies formulated by Wuhan and Hubei before the establishment of the headquarters.

Second, the 55 similar prevention and control policies made by different levels of government and different departments were discarded.

Ultimately, 67 prevention and control policies were discarded, and 445 policies were selected to reflect the Chinese government’s COVID-19 pandemic prevention, control, and management policies (Table 1).

As shown in the table, the central government level formulated the majority of the prevention and control policies (351, which accounted for 78.9% of the total). This result directly confirms the unified leadership and command prevention and control strategy of the Chinese central government during the COVID-19 pandemic. In terms of policy formulation, the central government played an obvious leading role in the design and formulation of COVID-19 pandemic prevention and control policies compared to the provincial and municipal governments. The central-level policy sources referred to in this paper include the important speeches and conference statements made by Xi Jinping and the prevention and control policies issued by the Leading Group of the CPC Central Committee for Novel Coronavirus Prevention and Control, the Central Political and Legal Committee, the Central Military Commission, the Joint Prevention and Control Mechanism of the State Council, and the ministries and commissions of the State Council. Among them, the three ministries and commissions of the State Council with the highest number of selected prevention and control policies were the National Health Commission (NHC), the Ministry of Transportation, and the Ministry of Education, with 115, 37, and 14 policies, respectively.

### 3.2. Methods

The 445 policies were first preprocessed with text separation and text feature extraction by ROST. Then, all the policies were analyzed by VOSviewer software for word occurrence statistics and econometric visualization [61]. Additionally, we categorized and analyzed the high-occurrence words. Finally, based on the results of policy measurement and visualization, we systematically studied the characteristics of policies and political institutions during the pandemic prevention and control governance. Specifically, the research methodology can be divided into two steps.

#### 3.2.1. Policy Preprocessing and Visualization

All prevention and control policies were preprocessed by ROST, which is large-scale data-mining software developed by Professor Shen Yang’s team at Wuhan University, China to assist researchers in the humanities and social sciences. Since both the classification corpus and the test corpus of ROST experiments are in Chinese, the Chinese preprocessing ability of the software is strong, but the classification algorithm is relatively simple [62]. Therefore, in this paper, ROST was chosen to preprocess the policies by mainly using the processes of text separation and text feature extraction. Afterwards, the word occurrence statistics, co-occurrence network analysis, and visualization of the thematic keywords of the policies were conducted by VOSviewer software [63], which generated the word occurrence table, co-occurrence network, and visualization. Then, we further categorized and analyzed the keywords, and, based on the co-occurrence network and visualization, we performed an in-depth analysis of the reasons for and effects of frequent mentions of institutional strengths.

#### 3.2.2. From Institutional Strength to Effective Governance: A Logical Reasoning and Approach Clarification

Through the measurement and visualization analysis of China’s pandemic prevention and control policies, we systematically present the institutional strengths relied on by the Chinese government in the process of policy formulation and subsequent implementation. Then, we explore the evolutionary mechanism and interaction logic between institutional strengths and effective governance in the COVID-19 pandemic prevention and control process. Then, by combining theoretical science in the field of public management and governance policy practice in China’s pandemic prevention and control, an in-depth integration analysis is conducted to clarify the approaches for transforming China’s institutional strengths into effective governance.

## 4. Results

The results of the study mainly include (1) policy word occurrences, (2) the use of institutional strengths and their visualization, and (3) the relationship between institutional strength and governance effectiveness.

### 4.1. Policy Word Occurrences

First, the word occurrences in the policy preprocessing data were determined by VOSviewer software, and the statistics obtained on the top 75 words are shown in Table 2, below. To better analyze the keywords, we categorized the top 75 high-frequency keywords according to the lexical meaning of each keyword and the semantics in the policy text where they are located. We found that, except for COVID-19 pandemic-related medical terminology, the rest of the keywords had obvious policy attributes and orientations, such as the specific content of the COVID-19 prevention and control policy, the actors in policymaking and implementation, and the requirements of policy implementation. Therefore, we organized the top 75 high-frequency keywords into 6 major categories (Table 3). “Institutional strength” refers to the distinctive strengths of the Chinese political system as noted by the Chinese government in the 4th Plenary Session of the 19th Central Committee of the CPC. “Medical terminology” refers to all medical terms related to COVID-19. “Policy content” refers to the specific content of the prevention and control policy, such as specific policy measures regarding transportation and material supply. The “policy implementation object” is the object of prevention and control policy implementation, such as people, communities, or businesses. “Policy implementation requirements” refer to the requirements and specifications of policy implementation, such as the strict implementation of isolation, strengthening scientific prevention and control, and comprehensive disinfection. “Policymaking and implementation actors” refer to the formulators of prevention and control policies, including General Secretary Xi Jinping and various government departments at all levels.

**Figure 3:**
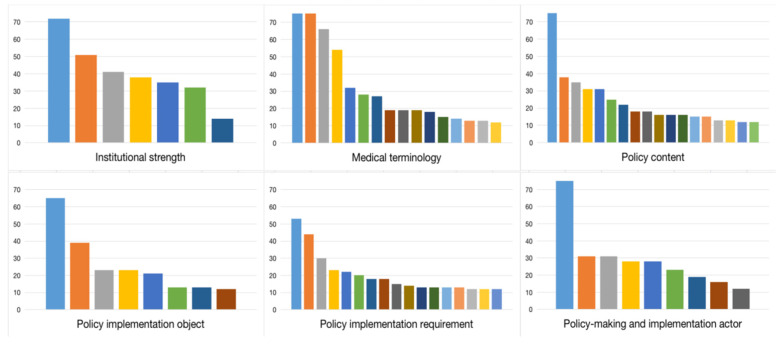
Word occurrences in the six categories. Source: Authors’ compilation. Note: the different color bars refer to the occurrences of each keyword in Table 3. For example, in the top left bar chart, the seven colors of light blue, orange, gray, yellow, blue, green, and dark blue (from left to right) represent the seven-word occurrences of “law-based governance”, “four confidences”, “armed forces”, “CPC centralized and unified leadership”, “the whole country working together”, “people-centered”, and “relying on people” (from high to low) in the second row (the category of institutional strength) of Table 3.

The Chinese government has repeatedly emphasized giving full play to China’s institutional, political, and organizational strengths in the formulation of COVID-19 prevention and control policies and governance logic. Correspondingly, the categorization of the institutional strengths of “law-based governance”, “four confidences”, “armed forces”, “CPC centralized and unified leadership”, “the whole country working together”, “people-centered”, and “relying on people” were referred to 72, 51, 41, 38, 35, 32, and 14 times, respectively. The occurrences of these keywords were much higher than the average occurrences of other keywords (Figure 3), except for the keywords that have the lexical meaning of the pandemic itself, such as “pandemic”, “prevention and control”, “health”, and “Xi Jinping” in the other categorization figures.

### 4.2. Use and Visualization of Institutional Strengths

This section addresses the use of institutional strengths and the presentation of the overall network and density visualizations of the COVID-19 pandemic prevention and control policies in China.

First, seven keywords—“law-based governance”, “four confidences”, “armed forces”, “CPC centralized and unified leadership”, “the whole country working together”, “people-centered”, and “relying on people”—were frequently mentioned. These keywords were merely from the 13 institutional strengths of China, as stated in the 4th Plenary Session of the 19th Central Committee of the CPC (Table 4). The Chinese government and scholars commonly agree that these thirteen strengths are the fundamental prerequisites for China’s political institutions, and they allowed China to create an “economic growth miracle” and maintain social stability [64].

The seven institutional strengths are applied simultaneously to a single governance scenario, which is the first case in China. The Chinese government repeatedly mentioned and emphasized seven institutional strengths which they can be better translated into effective governance for COVID-19 pandemic prevention and control [65,66]. The other six institutional strengths were not as frequently mentioned because they had very low relevance to the prevention and control of COVID-19. These strengths included “upholding equality between all ethnic groups”, “pursuing constant self-development”, and “upholding the principle of ‘one country, two systems’”. These unmentioned institutional advantages do not contribute much to effective governance in terms of prevention and control.

**Figure 4:**
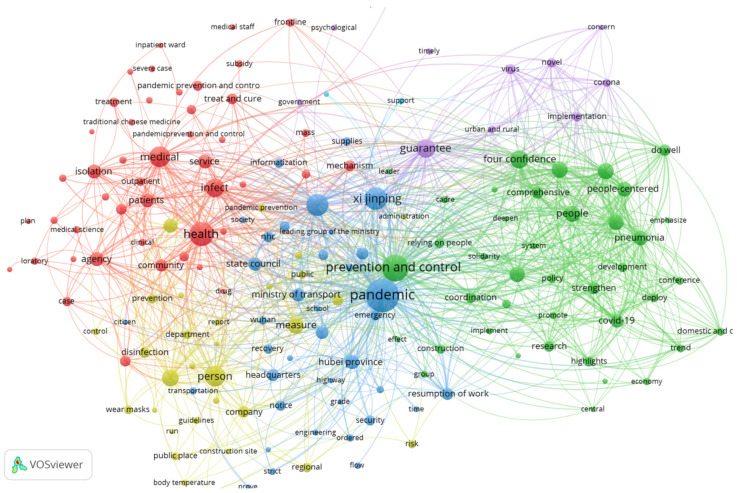
Network visualization of Chinese COVID-19 pandemic prevention and control policies. Source: Generated by the authors.

Second, VOSviewer was used to analyze and visualize the co-occurrence network of keywords in the policies, and the network visualization results are shown in Figure 4. The figure shows that institutional strengths, such as “law-based governance”, “four confidences”, “armed forces”, “CPC centralized and unified leadership”, “the whole country working together”, “people-centered”, and “relying on people”, have a significant intensity of connection with “pandemic” and “prevention and control”. This group of green and blue elements that represent China’s institutional strengths was clearly important in the policies.

**Figure 5:**
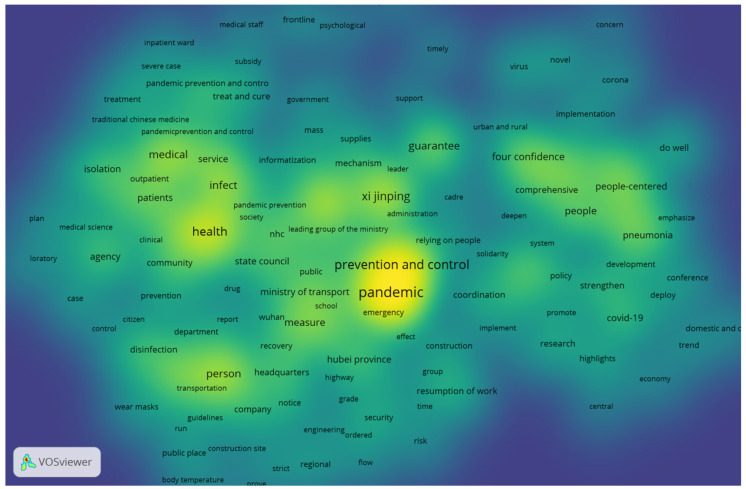
Density visualization of Chinese COVID-19 pandemic prevention and control policies. Source: Generated by the authors.

The density visualization also indicates that the density and importance of keywords in the category of “institutional strength” were second only to those of “pandemic” and “prevention and control” (Figure 5). Institutional strength was the logical premise of and a key factor in the Chinese government’s prevention and control policies. Therefore, the underlying logic of the Chinese government is to fully rely on China’s institutional strengths and to translate them into real effective governance in COVID-19 pandemic prevention and control.

Among these keywords, which reflect the strengths of China’s institutions, “law-based governance” appeared the most frequently, with the highest number of links and total link strength reaching 132 and 570, respectively (Table 5). This result is in line with the fact that, in recent years, the Chinese government has identified the rule of law as the basic strategy for governing the country, the fundamental way to solve major social problems, and a key requirement for liberating and enhancing social vitality to promote social justice, maintain social harmony and stability, and ensure the long-term stability of the Party and the country. The Chinese government’s adoption of “law-based governance” as the fundamental principle and basic guideline for the formulation and implementation of COVID-19 pandemic prevention and control policies is an important reason for the significant strategic results achieved and the rapid realization of effective governance.

Interestingly, the occurrences, links, and total link strength of “four confidences”, “armed forces”, “CPC centralized and unified leadership”, “the whole country working together”, and “people-centered” are very close to one another. As a result, the probability is that these keywords will appear together in the prevention and control policies, which is confirmed in the relevant policies. For example, in the *Work Plan for the Prevention and Control of Novel Coronavirus Infection and Pneumonia* formulated by the Joint Prevention and Control Mechanism of the State Council on 27 January, “four confidences” and “the whole country working together” each occur once. From this perspective, the institutional strengths of these five keywords are important for the effectiveness of COVID-19 pandemic prevention and control.

Finally, the occurrences, links, and total link strength of “relying on people”, at 14, 72, and 147, respectively, had the lowest values. This result is because “relying on people” is fundamentally different from the other six keywords, which reflect the strengths of institutions. “Relying on people” refers to treating people as leaders and promoting public cooperation through active interaction with people [67] to achieve effective governance in COVID-19 pandemic prevention and control. The lower occurrence of “relying on people” compared to the other six keywords is observed because the other six keywords are more focused on the methods of COVID-19 pandemic prevention and control, which makes them instrument-oriented.

### 4.3. The Relationship between Institutional Strength and Governance Effectiveness

From the policy analysis of China’s governance practice of COVID-19 pandemic prevention and control, institutional strength is a basic condition for achieving prevention and control goals. With its fundamental institutional strength, the Chinese government has formulated a series of COVID-19 pandemic prevention and control policies [68,69] and has frequently mentioned and emphasized the keywords of institutional strength such as “law-based governance”, “four confidences”, “armed forces”, “CPC centralized and unified leadership”, “the whole country working together”, “people-centered”, and “relying on people”. This has led to the two-way evolution and interaction of “institution policy” and supported strategic effective governance in COVID-19 pandemic prevention and control. Thus, the achievement of COVID-19 pandemic prevention and control goals and the emergence of effective governance is the logical continuation of institutional strengths [70,71].

First, institutional strength is a prerequisite for achieving effective governance. Whether effective governance can be achieved depends on the institutional form and system in a specific environment [72]. Institutional strengths arise from a series of effective institutional forms and systems that can fully interoperate with the governance structure, fit into the existing governance model, and adapt to the existing governance system [73]. Once institutions have strengths, they can promote coherence in the “means-goal” relationship of public governance and be consistently implemented [74], which achieves effective public governance. China’s prevention and control governance practices in response to COVID-19 show that the Chinese government has fully capitalized on the institutional strengths that have evolved endogenously through long-term development and gradual improvement [75] and that include “law-based governance”, “four confidences”, “armed forces”, “CPC centralized and unified leadership”, “the whole country working together”, “people-centered”, and “relying on people”. These factors made the COVID-19 pandemic prevention and control policies formulated by the Chinese government more feasible and effective. Then, through resolute and strong policy implementation and strict oversight, effective governance was achieved in a short time, and a decisive victory was achieved in the battle for COVID-19 pandemic prevention and control. Therefore, institutional strengths are a prerequisite for the rapid and effective governance of China’s COVID-19 pandemic prevention and control.

Second, effective governance is the logical continuation of institutional strength. From China’s practice of prevention and control, we can see that the relationship between institutional strength and effective governance is not simply causal. Although institutional strength is an important antecedent of effective governance, it also reacts to institutional strength; it is the logical continuation of institutional strength and an important condition for enhancing it. The formation of institutional strength facilitates the better functioning of the institution, which makes effective governance gradually apparent.

## 5. Discussion and Implications

Institutional strength and effective governance can be realized only by the Chinese government (not the market or business) through national scientific policy design. The results show that, except for medical terminology regarding COVID-19, the rest of the keywords relate to institutional advantage or policy formulation and implementation; that is, institutional strengths are translated into effective governance through policy formulation and implementation. Meanwhile, strict oversight is also essential, and the lower frequency of oversight keywords is because the Chinese government first focused on controlling the pandemic in the early and middle stages, with oversight also playing an important role in the later stages.

### 5.1. Scientific Policymaking: Public Value Integration

The government should focus on the integration of citizen values and scientific values in the policymaking process. Classical policy science theory posits that public policy formulation should be based on the pursuit of citizen values and that the role and status are the same as the scientific, economic, cultural, and social factors that affect policy formulation, such that science need not be given a higher priority or special status. In China’s response to COVID-19, decision-makers in the prevention and control of the pandemic were confronted with the interaction of civic and scientific values at different stages.

First, during the early stage of the COVID-19 pandemic, i.e., from the emergence of COVID-19 cases until 19 January 2020, the Chinese government showed a relatively strong pursuit of citizen interest-based values in the prevention and control policymaking process. At this stage, as the scientific knowledge of COVID-19 was still relatively superficial and the “two sessions” were held in Hubei and Wuhan, the primary theme of the prevention and control policies formulated by the governments of Hubei and Wuhan was the stable development of civic values and public economic and social order. Representative incidents included the warning and reprimand of Dr. Li Wenliang, who disclosed COVID-19 in advance, for “posting inaccurate statements on the internet” and notifications of “no obvious human-to-human transmission or infection of medical personnel” and “preventable and controllable” disease transmission.

Second, in the middle and late stages of the pandemic, i.e., after 20 January 2020, as scientific research on COVID-19 progressed, the balance of the pandemic prevention and control policy gradually began to tilt toward scientific factors, and the prevention and control of the pandemic were gradually dominated by “scientific values”.

The logical premise of the shift from “civic values” to “scientific values” is the gradual deepening of scientific research on COVID-19. This result pushed governments at all levels to realize that the civic values of social and economic stability must give way to the prevention and control of the COVID-19 pandemic. This change imposed enormous political pressure on the governments of Hubei and Wuhan to prevent and control COVID-19. Thus, they began to adopt a series of large-scale prevention and control policies, including the “city lockdown”.

The process of pandemic prevention and control policies in the two different periods clearly indicates that the Chinese government did not simply separate “civic values” and “scientific values”. Instead, it continually promoted their organic integration and negotiation, with “scientific values” gradually increasing and dominating policy decisions. This is an important reason why the Chinese government was able to achieve decisive results in the prevention and control of COVID-19 within three months. This finding contradicts the supposition of most Western scholars that “scientific factors” are less important and discursive in the public policymaking process in centralized, unitary China [76].

### 5.2. Efficient Implementation: Pressurized System and High-Level Promotion

The concept of a pressurized system, which was first proposed by Chinese scholar Rong Jingben [77], is considered a special context of public policy implementation in China, different from that of developed Western countries. The core of the pressurized system is “politicized implementation”, which is defined as a “political task” by higher-level governments or officials to ensure that lower-level governments or officials complete a specific task with high quality. This pressure usually occurs under the command of a “top leader”, and those who fail to complete the task face punishment by power means, such as “veto power” [78]. High promotion refers to the transmission of pressure from higher levels of government or officials to promote policy implementation that is more efficient in the context of socialism with Chinese characteristics. The underlying reason for this situation is the logic of the top-down political resource allocation within the Chinese public governance system in terms of hierarchy, pressure transmission, disciplinary monitoring, and power tools. In this process, government departments or officials at higher levels achieve better results from pressure transmission and responsibility implementation [79]. Thus, policy implementation tends to be more effective when driven from a high level.

The prevention and control policies show that the effective governance of policy implementation is highly driven by the pressurized Chinese system. The recurrence of the pressurized system attributes represented by the keywords “political stand”, “political assurance”, “political task”, “political quality”, and “serious accountability” put the government at all levels under strong political pressure to prevent and control the COVID-19 pandemic and implement the policies of higher-level government with “political implementation” [80]. This phenomenon also suggests that the pressurized Chinese system has become an important motivator for government and officials at all levels to promote the implementation of prevention and control policies. Under the pressurized system, Chinese government officials rely not only on traditional incentives, such as the rule of law, economics, and power, but also on their commitment and devotion to the organization’s specific political beliefs under the strong ideology of the CPC. This situation is in line with Rothstein’s suggestion that the Chinese government has a special “cadre organization” management model that is distinct from Weberian bureaucracy [81].

In addition to the efficient implementation of the pressurized system, the high status of the Party and government leaders in high positions in the pressurized system is an important basis for the efficient implementation approach of the COVID-19 pandemic prevention and control policies, which is exactly what the pressurized system implies. High-level promotion has been fully utilized in the prevention and control of COVID-19. The top leaders of the Chinese central government, which is represented by General Secretary Xi Jinping and Premier Li Keqiang, have attached great importance to the prevention and control of COVID-19, listened to many reports on the subject, directly commanded personnel and deployed resources, and requested the “top leaders” of the Party and government at all levels to actively deploy and fully concentrate on pandemic prevention and control. These actions form a “multilayered, high-level promotion” model of government at all levels, which results in leadership-led departmental coordination, financial support, and results-oriented COVID-19 pandemic prevention and control mechanisms through the “command and control system” led by “high-level leadership” [82]. Specifically, for COVID-19 pandemic prevention and control, being leadership-led means that the top leader of the Party and government in the local region is the first person responsible for the implementation of COVID-19 pandemic prevention and control policies and is the head of the local pandemic prevention and control leading group. Departmental coordination means that the top leader of the Party and government at all levels convenes the heads of affiliated functional departments to clarify the responsibilities, rights, and obligations of the multifunctional actors in COVID-19 pandemic prevention and control. Financial support refers to the allocation of special funds for COVID-19 pandemic prevention and control to ensure the funding required for the implementation of prevention and control policies. Being results-oriented refers to the “political task” and target assessment accountability system under the pressurized system and bureaucratic model to restrain the implementation of prevention and control policies so that government officials hold their positions, take the lead, and carry out their duties toward all people.

### 5.3. Strict Oversight: Effective Integration of CPC Internal and External Oversight

Samuel P. Huntington believes that oversight is an important means of curbing the corruption of government officials and that only by increasing oversight and restraining power can the exercise of power be regulated and controlled. The developing practices of the CPC in the past century have also shown that strengthening oversight plays a key role in realizing the Party’s self-purification, regulates the behavior of Party cadres, improves the Party’s governing ability, and promotes its development and growth [83]. Since the 18th National Congress of the CPC, the Party’s internal oversight approach has made notable advancements in building effective power oversight and discipline enforcement systems. At the 19th National Congress of the CPC, the Chinese government further identified “improving the oversight system of the Party and the State” as a major task of Party construction in the new era. At this time, the government clearly proposed “establish[ing] an authoritative, efficient oversight system with complete coverage under the Party’s unified command; and integrat[ing] internal Party oversight with oversight by State organs, democratic oversight, judicial oversight, public oversight and public opinion oversight to create a powerful synergy for conducting oversight” [84]. At the 4th Plenary Session of the 19th Central Committee of the CPC, China once again proposed an important strategic plan to improve the oversight system of the Party and the State [85]. This decision provided a conceptual guideline and path for the Chinese government to adhere to and improve the oversight approach of the Party and the State at this stage of the pandemic.

In China’s current Party and State oversight system, the Party’s internal oversight is dominant. General Secretary Xi Jinping pointed out that among the various forms of Party and State oversight, the Party’s internal oversight is the most fundamental and important [86]. However, the role and status of external oversight in China should not be underestimated. The Regulations of the CPC on Internal Oversight devote an entire chapter to a detailed discussion of the importance and necessity of insisting on the combination of the internal and external oversight of the Party [87]. In terms of role and status, the CPC’s external oversight can be divided into two categories. The first is oversight by State organs, which is power oversight in nature and refers to oversight by departments with the legal oversight power of the State, which mainly includes oversight by State power organs, judicial oversight, administrative oversight, etc. The second category is social oversight, which is rights oversight, that is, oversight by the people as individuals or mass organizations, social organizations, news media, and other nonstate oversight institutions. This category mainly includes the oversight of the public, mass organizations, democratic parties, and public opinion. China has established a comprehensive, well-structured, and smoothly functioning oversight approach through the effective integration of the CPC’s internal and external oversight systems [88].

During the COVID-19 pandemic prevention and control period, China’s oversight approach showed the typical characteristics of the effective integration of internal and external oversight by the CPC. Additionally, the structural shape of the integrated oversight approach was more organic and tighter. This approach made good use of the overall advantages of oversight and supported the implementation of pandemic prevention and control policies.

In terms of internal party oversight, the Discipline Inspection Commission (DIC) of the CPC at all levels formed a vertical linkage system and weaved a tight and solid COVID-19 pandemic prevention and control oversight network. From the perspective of China’s current administrative system, the colocation of the DIC and the State Supervisory Committee (SSC) of the CPC allows them to perform both disciplinary and inspection functions, i.e., “two departments with one set of personnel”. Thus, the internal oversight of the Party, the oversight of State organs, and the disciplinary inspection of the Party and State oversight are organically unified to ensure the full oversight of all public officials who exercise public power.

Under the COVID-19 pandemic prevention and control oversight network, many local DICs have set up supervisory and inspection task forces and established mechanisms for the quick referral and investigation of pandemic prevention and control issues to promote the layered implementation of the work of COVID-19 pandemic prevention and control [89]. The Chinese government drives the implementation with accountability by quickly and decisively taking measures against the CPC members and cadres who do not actively take charge but cope only passively. In Hubei Province, more than 3000 CPC members and cadres were punished for failing in their duties and responsibilities during the critical period of prevention and control in April, and more than 10 CPC members and cadres at the department levels were punished [90]. Compared with the historical practice of oversight in China, the speed, strength, and severity of the prevention and control accountability led by the DIC in COVID-19 are exceptional.

External Party oversight—as represented by the National People’s Congress (NPC), the judiciary, and the SSC—is exercised over the main body of power and ensures that all State organs and their staff use their power legally and compliantly in matters regarding COVID-19 pandemic prevention and control. In the case of the NPC and its Standing Committee, for example, oversight mainly includes legal and work oversight. Regarding legal oversight, the central and local people’s congresses provide important safeguards for emergency legislation and oversight for COVID-19 pandemic prevention and control. These bodies urgently issue laws and regulations and special regulatory decisions, such as the *Decision on Comprehensively Banning the Illegal Wildlife Trade, Eliminating the Bad Habit of the Indiscriminate Consumption of Wildlife, and Effectively Safeguarding People’s Lives*; *Regulations on the Management of Experimental Animals*; and *Emergency Measures for Public Health Emergencies in Guangdong Province*. These measures were taken during the critical period to create timely and powerful legislative safeguards for pandemic prevention and control. Regarding work oversight, in addition to the cooperation of the NPC at all levels with national law enforcement inspections, the local people’s congresses of Guangxi, Shandong, and other provinces are dedicated to the implementation of the Emergency Response Law. Moreover, the local people’s congresses of Liaoning, Jiangsu, and other provinces have carried out regional law enforcement inspections under the *Drug Administration Law*.

## 6. Conclusions

Public health crises pose tremendous risks to human life and health and social and economic development. This paper empirically examines the characteristics of policies and political institutions for the prevention and control governance of the COVID-19 pandemic in China through an econometric visualization analysis. The results show that (1) the Chinese government has made full use of seven institutional strengths and (2) their occurrences, links, and total link strength are very high. The policy analysis result suggests that China’s institutional strength is the basic condition for achieving effective governance; accordingly, we systematically analyzed the approaches to realizing effective governance—which include scientific policymaking, efficient implementation, and strict oversight—thereby providing a reference for the governance of public health crises in other countries.

However, certain shortcomings also exist. The first is the specificity of the research object. The results and conclusions are likely to vary somewhat regarding public governance objects or behaviors in China other than those regarding the prevention and control of the COVID-19 pandemic. The second limitation is the research method. Due to space limitations, this paper simultaneously analyzes all the policies of different government levels and does not distinguish them by government level, so horizontal variability may be omitted. These two shortcomings offer directions for further research.

## Figures and Tables

**Table 1:** Selected data on the COVID-19 pandemic prevention and control policies from three levels of government in China.

Policy Formulation Actors	Scope of Actors	No. of Policies	Percentage
Chinese Central Government, State Council	Xi Jinping, Leading Group of the CPC Central Committee for Novel Coronavirus Prevention and Control, Central Political and Legal Committee, Central Military Commission, Joint Prevention and Control Mechanism of the State Council, and Ministries and Commissions of the State Council	351	78.9%
Hubei Province	Hubei Province COVID-19 pandemic prevention and control headquarters	31	7.0%
Wuhan City	Wuhan City COVID-19 pandemic prevention and control headquarters	26	5.8%
Beijing City	Beijing municipal commissions and bureaus	37	8.3%
Total		445	100%

Source: Authors’ compilation.

**Table 2:** Word occurrence statistics in the COVID-19 pandemic prevention and control policies (*n* = 75).

No.	Keyword	Occurrences	No.	Keyword	Occurrences	No.	Keyword	Occurrences
1	Pandemic	170	26	Ministry of Transport	28	51	Emergency	15
2	Prevention and control	108	27	State Council	28	52	Highlights	15
3	Health	87	28	Patients	27	53	Security	15
4	Xi Jinping	80	29	Transport	25	54	Virus	15
5	Law-based governance	72	30	Community	23	55	Novel	14
6	Medical	66	31	Comprehensive	23	56	Relying on people	14
7	Person	65	32	Enterprise	23	57	Science	14
8	Infect	54	33	Headquarters	23	58	Case	13
9	Guarantee	53	34	Resumption of work	22	59	Community-level	13
10	Four confidences *	51	35	Strengthen	22	60	Construction	13
11	Management	44	36	Company	21	61	Development	13
12	Armed forces	41	37	Do well	20	62	Implementation	13
13	People	39	38	Disinfection	19	63	Informatization	13
14	Measure	38	39	Fever	19	64	Prevention	13
15	CPC centralized and unified leadership	38	40	NHC	19	65	Recovery	13
16	Economic and social development	35	41	Testing	19	66	Regional	13
17	The whole country works together	35	42	Coordination	18	67	Treatment	13
18	People-centered	32	43	Mechanism	18	68	Department	12
19	Pneumonia	32	44	Research	18	69	Deploy	12
20	Agency	31	45	Work and production resumption	18	70	Guidance	12
21	Hubei Province	31	46	Treat and cure	18	71	Outpatient	12
22	Protective	31	47	Pandemic prevention and control	16	72	Policy	12
23	Service	31	48	Normalization	16	73	Resolute	12
24	Isolation	30	49	Notice	16	74	Supplies	12
25	COVID-19	28	50	Party central committee	16	75	Wear masks	12

Source: ROST and VOSviewer software. * Note: The four confidences refer to confidence in the path, theory, system, and culture of socialism with Chinese characteristics, which was proposed by General Secretary Xi Jinping at the conference celebrating the 95th anniversary of the CPC founding.

**Table 3:** Categorization of the top 75 high-frequency keywords in the prevention and control policies.

No.	Category	Keywords
1	Institutional strength	law-based governance, four confidences, armed forces, CPC centralized and unified leadership, the whole country works together, people-centered, relying on people
2	Medical terminology	pandemic, health, medical, infect, pneumonia, COVID-19, patients, disinfection, fever, testing, treat and cure, virus, novel, case, treatment, guidance
3	Policy content	prevention and control, measure, economic and social development, protective, service, transportation, resumption of work, mechanism, resumption of work and production, pandemic prevention and control, normalization, notice, emergency, highlights, construction, development, policy, supplies
4	Policy implementation object	person, people, community, enterprise, company, community-level, regional, outpatient
5	Policy implementation requirement	guarantee, management, isolation, comprehensive, strengthen, do well, coordination, research, security, science, implementation, informatization, prevention, recovery, deploy, resolute, wear masks
6	Policy-making and implementation actor	Xi Jinping, agency, Hubei province, Ministry of Transport, State Council, headquarters, NHC, Party Central Committee, department

Source: Authors’ compilation.

**Table 4:** Institutional strengths in COVID-19 prevention and control policies..

No.	China’s Thirteen Institutional Strengths	Mentioned or Not	Key Relevant Topic Words or Phrases in the Policies
1	Upholding the four confidences	√	Upholding the four confidences
2	Upholding CPC’s centralized and unified leadership	√	Upholding CPC’s centralized and unified leadership Upholding Party centralized and unified leadership
3	Relying on people to drive national development	√	Relying solely on the people
4	Pursuing law-based governance in all respects	√	Orderly prevention and control based on law and science Prevention and control by law Law-based governance, implementation of measures by law Pandemic information release must be prescribed by law Support and cooperation with pandemic prevention and control by law Equal protection of the legitimate rights and interests of all types of market subjects by law Performance of procuratorial oversight by law Strengthening oversight and enforcement by law Law-based oversight
5	Upholding the whole country working together	√	Upholding the whole country working together Establishing the mindset of the whole country working together
6	Upholding equality between all ethnic groups	/	/
7	Creating and unlocking additional productive forces	/	/
8	Uniting the nation in shared beliefs and convictions	/	/
9	Pursuing a people-centered approach	√	Pursuing a people-centered approach Firmly pursuing a people-centered approach to development
10	Pursuing constant self-development	/	/
11	Selecting the best minds across the land	/	/
12	Ensuring the people’s armed forces’ absolute loyalty to the Party and the people	√	Armed forces actively support the prevention and control of the COVID-19 pandemic Remembering the purpose of the people’s armed forces Transferring medical forces from armed forces Armed forces’ absolute loyalty to the Party and the people People’s armed forces are heroic and can be fully trusted by the Party and the people Relevant health departments of the armed forces
13	Upholding the principle of “one country, two systems”	/	/

Source: The 13 institutional strengths come from the 4th Plenary Session of the 19th Central Committee of the CPC. Note: “/” indicates that the Chinese government did not mention this institutional strength as a keyword in a COVID-19 pandemic prevention and control policy.

**Table 5:** Keyword statistics of the institutional strengths employed in the COVID-19 prevention and control policies.

No.	Item	Occurrences	Links	Total Link Strength
1	Law-based governance	72	132	570
2	Four confidences	51	102	553
3	Armed forces	41	94	452
4	CPC centralized and unified leadership	38	85	465
5	The whole country works together	35	86	440
6	People-centered	32	83	405
7	Relying on people	14	72	147

Source: Authors’ compilation.

## Data Availability

Not applicable.
